# Editorial: Cell compartments and intracellular trafficking of lipids and proteins: Impact on biomedicine

**DOI:** 10.3389/fcell.2022.1087214

**Published:** 2022-11-24

**Authors:** Silvana Zanlungo, Carlos Enrich, Volker Gerke, Emily R. Eden, María Isabel Colombo

**Affiliations:** ^1^ Department of Gastroenterology, Faculty of Medicine, Pontificia Universidad Católica de Chile, Santiago, Chile; ^2^ Department of Biomedicine, Unit of Cell Biology, Faculty of Medicine and Health Sciences, Centre de Recerca Biomèdica CELLEX (IDIBAPS), University of Barcelona, Barcelona, Spain; ^3^ Institute of Medical Biochemistry, Centre for Molecular Biology of Inflammation, University of Münster, Münster, Germany; ^4^ UCL Institute of Ophthalmology, London, United Kingdom; ^5^ Laboratorio de Mecanismos Moleculares Implicados en El Tráfico Vesicular y la Autofagia, Instituto de Histología y Embriología de Mendoza (IHEM), Universidad Nacional de Cuyo- Consejo Nacional de Investigaciones Científicas y Tecnológicas (CONICET), Mendoza, Argentina

**Keywords:** Endosomes, lysosomes, membrane contact sites, lysosome-related organelles, cholesterol, lysosomal storage diseases, disease models, lipid droplets

Endocytosis and cell compartments define eukaryotic cells and evolution has sculpted a complex subset of intracellular structures to arrange the modern cell. This process has been long, implicating each single organelle to eventually develop a functional consortium where spatio-temporal distribution becomes essential.

Because of this intrinsic extreme complexity minor alterations must be repaired, replaced, or undergo reorganization. This is also important when it comes to biochemical processes within and between cellular compartments developing a new concept that implicates cell compartments not to function as individual and isolated entities, but as a dynamic and regulated ensemble facilitating the intracellular trafficking of lipids and proteins. The diverse intracellular compartments contribute to a global cell homeostasis and alterations provide new insights relevant for a number of human diseases and offer opportunities for the design of innovative therapies and treatments.

In recent years we increasingly appreciate the complex organization of the crowded intracellular space. Sorting and trafficking along major routes travelled by vesicles implicate maturation, fission, and fusion of membranes and the most universal means of achieving compartmentalization is probably by the self-assembly of membranes into units, the organelles. These units maintain specific form and composition and emerge from elaborate and coordinated membrane trafficking pathways that manifest themselves either as explicit membrane bound carriers or through regulated physical proximity of organelles (membrane contact sites). Indeed, the endoplasmic reticulum (ER) is the organelle “to rule them all”; it is the most extensive membrane compartment, lead regulator of membrane trafficking, the largest Ca^2+^ store and the compartment in charge for the biosynthesis of lipids, proteins and assembly of glycoconjugates (Wenzel et al., 2022).

The 14 articles in this collection cover only a small part of the wide panorama, but representative aspects are discussed to understand membrane trafficking across organelles from a wide biological, biophysical, or engineering perspectives and where protein sorting, membrane traffic, and organelle dynamics are the targets and alterations may be the cause of disease.

Endocytosis plays a key role in the regulation of signalling from cell surface receptors. In an original research article by Artyokov et al., endocytosis of death receptors is explored. Tumour necrosis factor (TNF)-associated ligand inducing apoptosis (TRAIL) binds cell surface death receptors DR4 and DR5, initiating a signalling cascade that results in apoptosis. Through unknown mechanisms, some cancer cells are resistant to TRAIL-induced apoptosis, limiting the anti-cancer potential. Here, Artyokov et al. determine the role of DR4 and DR5 endocytosis in conferring sensitivity to TRAIL-induced apoptosis. The authors demonstrate that TRAIL binding universally stimulated endocytosis of DR4 and DR5 in sensitive and resistant cells alike, concluding that sensitivity to TRAIL-induced apoptosis arises through post-endocytosis mechanisms.

Membrane lipid composition is a key determinant of endocytic traffic and receptor signalling and Hasegawa et al. review the importance of endosomal phosphatidylserine (PS) distribution on trafficking pathways. PS is the major anionic phospholipid in the plasma membrane but is also enriched on recycling endosomes where it recruits proteins involved in recycling and retrograde transport for cargo delivery to the plasma membrane and Golgi respectively. Here Hasegawa et al. discuss the role of PS flipping from luminal to cytosolic leaflets of recycling endosomes by the P_4_-ATPase ATP8A in recycling and retrograde traffic. PS flipping is likely to be coordinated with the recently identified transport mechanism of newly synthesized PS from the ER to recycling endosomes at ER:endosome contact sites to promote recycling and retrograde transport (Kawasaki et al., 2022). Endosomal PS has also been implicated in YAP signalling, that regulates cell proliferation (Matsudaira et al., 2017), further demonstrating the importance of endosomal PS in membrane traffic and cell fate and Hasegawa et al. consider the potential significance of ATP8A1 mislocalisation in YAP signalling and Hermansky-Pudlak syndrome (HPS). On loss of the adaptor protein AP-3, that is defective in HPS, ATP8A1 accumulates on recycling endosomes, increasing the cytosolic exposure of PS, activating YAP and promoting cell division and migration.

The Research Topic continues to highlight the significance of the lipid environment in membrane traffic and disease. The importance of cholesterol transport and distribution inside of cells is a recurring theme (Ikonen, 2008; Lu, 2022). Here, a comprehensive review dissects the pathways for cholesterol efflux highlighting the state-of-the-art technology (Juhl and Wüstner). In addition, two original research articles study the role of cholesterol uptake in two pathogenic protozoa during infection. In *Toxoplasma gondii*, the relevance of cholesterol transport (Croce et al.) is underpinned using the cholesterol transport inhibitor U18666A demonstrating the potential of cholesterol for the recruitment of CHMP4, in MVBs formation, for optimum antigen presentation and parasite replication. Other related research (Okamoto et al.) addresses the problem of cholesterol uptake, in the pathogenic yeast *Candida glabrata*, as strategy to decrease antifungals susceptibility for proliferation and the possibilities of using ERG25 in the stabilization of sterol-rich lipid domains as therapy.

The family of low-density lipoprotein receptors (LDLR) plays a key role in cholesterol internalization and homeostasis (Go and Mani, 2012). This family comprises plasma membrane receptors that bind several unrelated ligands, which are subsequently endocytosed. Megalin is one of the members (Saito et al., 1994; Hussain et al., 1999), which has a critical function in recovering low molecular weight proteins, from the renal glomerular filtrate, (Leheste et al., 1999). Thus, alterations in the trafficking of this receptor may result in proteinuria (Marzolo and Farfan, 2011). In this collection, an original research article analyzes the post-transcriptional modulation of megalin in the Lowe Syndrome (LS) (Sandoval et al.). LS patients present mutations in a gene encoding a phosphatase known as OCRL1. Megalin is proteolytically cleaved in its ectodomain and in LS patients reduced levels of this megalin fragment are detected. In addition, the levels of the receptor in endocytic/recycling compartments increase in the proximal tubule renal cells. The authors show that silencing OCRL1 mimics the altered parameters found in LS patients with a significant decrease of megalin at the plasma membrane, indicative of a reduced recycling of the receptor. Furthermore, in these LS conditions the phosphorylation levels of megalin were reduced, explaining, in part, the altered recycling transport. Sandoval et al. also uncovered the role of insulin in the reduction of megalin phosphorylation and trafficking to the plasma membrane. The generation of this LS-mimicking cellular model may potentially help to develop novel therapeutic tools for this disease.

Lipid droplet (LD) landscape (Olzmann and Carvalho, 2019; Bosch et al., 2020) is also covered in this Research Topic emphasizing the great importance of LD as dynamic intracellular compartment found in most cells playing fundamental roles in lipid metabolism but having interactions and contacts with other organelles that remain elusive. Two reviews, one summarizing the major mechanisms of biogenesis and breakdown of LD (Fader Kaiser et al.) and a second review about LD in the nucleus and their function at this novel sub-world (McPhee et al.). Both articles discuss and dissect the molecular components to correlate with the involvement in human pathologies.

In the recent years the lysosome has emerged as an essential organelle for cellular homeostasis, being not only involved in the degradation of complex substrates but also able of sensing the nutrient environment and participate in signal transduction, thereby regulating fundamental processes such as cellular clearance and autophagy (Ballabio and Bonifacino, 2020). A review in this collection (Cabrera-Reyes et al.) focuses on lysosome homeostasis alterations in lipid-related disorders, particularly in prevalent diseases such as obesity and also in the less frequent Lysosomal Storage Diseases (LSDs), such as Niemann-Pick C (NPC) and Gaucher diseases and discusses the mechanisms involved in lysosomal alterations that are common among cells of metabolic tissues, including adipose tissue and the liver, which are primarily affected in these pathologies. An original research article of this collection (Marín et al.) explores the relevance of alterations in c-Abl tyrosine kinase signalling in the LSD Niemann-Pick type A (NPA), a fatal neurodegenerative disorder caused by the deficiency in acid sphingomyelinase (ASM) activity and characterized by an accumulation of sphingomyelin in lysosomes and dysfunction in the autophagy-lysosomal pathway. The results show the participation of c-Abl signalling in NPA neurodegeneration and autophagy-lysosomal alterations, supporting the potential use of c-Abl inhibitors for the clinical treatment of NPA patients.

Lysosome related organelles (LROs) are a unique class of intracellular compartments performing specialized functions in different types of cells. In vascular endothelial cells, the prominent LROs are Weibel-Palade bodies (WPBs). They serve as storage organelles for the blood clotting von-Willebrand factor and the leukocyte receptor P-selectin that are released *via* evoked exocytosis of WPBs following endothelial activation by inflammation or blood vessel damage (McCormack et al., 2017). The unique biogenesis and exocytotic response of WPBs are highlighted in one minireview of this series focussing on recent developments that identified factors involved in WPB maturation and WPB-actin as well as WPB-plasma membrane interactions in the course of exocytosis (Naß et al.). An original research article describes the interesting trafficking route of a vacuolar ATPase (vATPase) subunit on its way to maturing WPBs (Lu et al.). WPBs are acidic organelles, and the low intraluminal pH is required for the proper folding (tubulation) of VWF in the organelle. Recently, vATPase activity and the vATPase V0a1 subunit were reported to be required for proper WPB acidification and biogenesis of the organelle (Yamazaki et al., 2021; Terglane et al., 2022) and Lu et al. now extend this to another vATPase subunit, V0D1. Importantly, they show that this subunit is transported to WPBs in a manner requiring the HPS6 subunit of the endosomal BLOC-2 complex. Knockdown of HPS6 in primary human endothelial cells results in misshaped WPB and impaired VWF tubulation, a phenotype also seen in HPS6 deficient mouse endothelial cells. Lu et al. also report a direct interaction of the vATPase V0D1 subunit with HPS6 suggesting that the BLOC-2 complex is involved in transporting vATPase V0D1 from endosomes to WPBs for assembly of a functional vATPase in the limiting membrane of the organelle.

Macroautophagy is a degradative pathway that intersects with both the lysosomal and the proteosomal pathway to maintain cellular homeostasis by degrading and recycling critical components. Increasing evidence indicates that autophagy can be regulated, either in a positive or negative way, by the ubiquitin proteasome system, indicating that both systems function as an interconnected network (Korolchuk et al., 2010; Bustamante et al., 2018). An original research article of this series addressing this topic (Vargas et al.) identified an ER membrane protein called HERPUD1. Expression of a deletion mutant lacking the ubiquitin-like domain, critical for proteosomal degradation, negatively modulates autophagy. In addition, overexpression of this mutant leads to an increase in ER tubular stacks as well as an augment in the biogenesis of lysosomal vesicles. The authors propose that ER-lysosome intercommunication is promoted to favor cell survival under stress conditions, such as nutritional deficiency or certain drug treatments.

Finally, membrane contact sites (MCS) are now at the crest of the wave in cell biology (Prinz et al., 2020). The paradigm has changed, and compartments communicate by vesicular trafficking but also *via* dynamic close contacts with a plethora of tethers and molecular machineries in charge for transport and exchange of lipids, cholesterol, ions, and metabolites. MCS modulate endosome maturation, organelle positioning, metabolic platforms and for cellular homeostasis. A Mini Review in this collection (Enrich et al.) put together recent findings regarding a subset of annexins (Gerke et al., 2005) and discusses their multiple possibilities to regulate MCS dynamics, function and possible contribution to novel pathways providing new insights relevant for several human diseases and offering opportunities to design innovative treatments.

In summary, the present collection of research articles and reviews under this Research Topic highlights the advances in the field with novel insights to uncover the fine structure, distribution, and dynamics of molecular machineries and protein complexes in cell compartments. Throughout the collection, a relationship between the lipid environment and membrane trafficking/signalling pathways is emerging. The composition of membrane lipids is diverse and complex and key open questions remain about the maintenance of membrane lipid homeostasis and its influence over the endocytic pathway. A role for MCS in coordinating these dynamic and adaptable processes is becoming apparent but the picture in different physio/pathological situations is incomplete and key intracellular cholesterol transport mechanisms remain elusive. With improved understanding, new interventions to help in the cure of multiple human diseases become a possibility.
